# Effects of media-assisted therapeutic approaches on physical activity of obese adults: a systematic review

**DOI:** 10.1186/s12902-020-0505-x

**Published:** 2020-02-21

**Authors:** Alexandra Ziegeldorf, Petra Wagner, Hagen Wulff

**Affiliations:** Institute for Exercise and Public Health, Faculty of Sports Science, Leipzig University, Jahnallee 59, 04155 Leipzig, Germany

**Keywords:** Obesity, Therapy, Aftercare, Digital media, Physical activity, Moderate to vigorous physical activity, Adults

## Abstract

**Background:**

The number of patients with obesity continues to increase in our society. At the same time, digitalization defines our everyday life. Therefore, the question arises whether to use digital media for effective treatments against obesity. Aim of this review is a concise presentation of effects of media-assisted therapeutic approaches.

**Methods:**

A systematic literature research in multiple scientific databases, PubMed, Web of Science and Cochrane Library, was performed for literature published in the last 10 years (cut-off date 12.07.2017). Only randomized controlled trials using digital support for treatment and/or aftercare of obese adults aged between 18 to 70 years were included into the analysis.

**Results:**

Overall, 14 trials with data on a total of 4979 participants were included in this review. Generally, there are positive trends for increased moderate to vigorous physical activity by the use of digital media. A simultaneous usage of various digital media, which focus on important aspects of activity behavior, has proven to be effective. A combination of various digital media shows the greatest positive effect and could significantly increase physical activity. However, the biggest potential was found in mixed approaches combining digital devices and personal face-to-face support.

**Conclusions:**

Therapeutic approaches using digital media for supporting obesity treatment did not show superior benefit over traditional personal therapeutic methods. Nevertheless, using both methods together offered the greatest potential for successful obesity therapy. However, there is a backlog of transparency concerning information about the content of treatment. Furthermore, there is a lack of valid data about aftercare and follow-up.

## Background

The prevalence of overweight and obesity has trebled since 1975 [1], and poses an enormous risk of accompanying and secondary diseases to patients [2]. As a consequence, healthcare systems face substantial direct and indirect costs [3].

Main risk factors for overweight and obesity are low physical activity and high inactivity, i.e. physical activity with decent intensity under the recommended 150 min/week [4, 5].

There are many different therapeutic approaches used all over the world to reduce patients’ individual burdens and costs for health care systems [6, 7]. According to international guidelines of obesity treatment, all stationary and ambulatory therapeutic approaches aim to increase physical activity [8].

The use of digital media in therapy has been controversially discussed in the corresponding literature [9, 10]. On the one hand, many studies indicate that an increased use of digital media is associated with reduced physical activity and increased risk of overweight and obesity [11–13].

In connection with television-presupposed inactivity some meta-analyses report an increased overall mortality [14], cardiovascular mortality [15] and tumor presupposed mortality [16]. Similarly, meta-analyses report an increased morbidity due to metabolic syndrome [11, 17]. On the other hand, digitization offers great potential for therapeutic settings as they may benefit from the use of digital media [18]. Digital media guarantee patients a contemporary, daily access to therapy and may help to facilitate and support therapeutic home care as their use is not bound to any treatment facilities [19]. For therapeutic approaches focused on an obesity-related lifestyle modification, digital media are also used for adoption, maintenance and long-term implementation of a healthy behavior including regular physical activity [20].

Therapeutic approaches use different media. In the study by Cussler et al. [21] emails, chatrooms and platforms were used to support participants in increasing their physical activity (intensity). Furthermore, interactive TV shows [22], text messages [23] and telephone coachings (see also [24, 25]) were used for lifestyle modifications and, in this context, to increase physical activity (intensity). Another approach focused on the difficult access to on-site therapy, which particularly challenging in rural areas. Donelly et al. [26] used conference calls to provide intervention programs to patients who would otherwise not have been able to participate at all.

Overall, empirical findings of therapeutic effects tend to be heterogeneous in terms of the desired increase in physical activity.

In the context of ambulatory and stationary therapy optimization, it is key to determine the efficiency of media-assisted obesity treatment in enhancing physical activity in adults. Therefore, this systematic review aims at analyzing the efficiency of therapeutic approaches using media for obesity treatment in adults.

## Methods

**Fig. 1:**
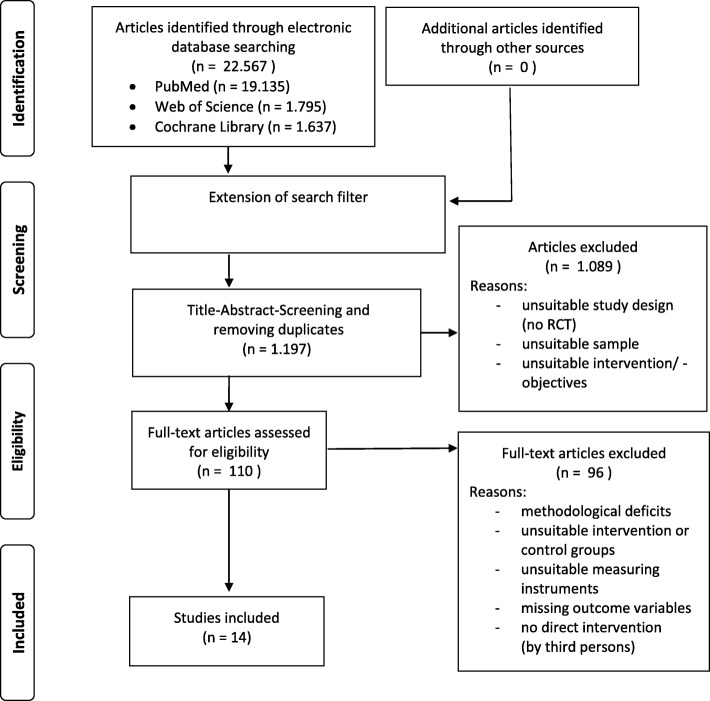
Flow chart diagram (PRISMA 2009) [28] describing selection of studies for the systematic review of Effects of media-assisted therapeutic approaches on physical activity of obese adults (identified, screened, eligible and included studies). Articles may have been excluded for more than one reason

A systematic literature review in multiple databases (PubMed, Web of Science and Cochrane Library) was performed for articles published from January 1st, 2007 to July 12th, 2017. The methodological approach of the review is based on the guidelines of the German Cochrane Community (2013) [27]. The procedure used in this study follows the PRISMA statement [28]. After identifying adequate keywords using the PICO scheme a corresponding search strategy was developed. After the first search and analysis of the results the strategy was modified. Following the final search, all relevant studies were examined and documented; duplicates were removed (Fig. 1). Inclusion criteria included all randomized controlled trials using digital media to support obesity therapy and/or aftercare. Included digital media were internet, PC, TV, video (games), DVD, mobile phone, smartphone, landline telephone and apps. All inclusion and exclusion criteria are listed below.

### Inclusion criteria

- age 18–70
- randomized controlled trials (RCT)
- direct intervention with digital media
- body mass index (BMI) at least 25 kg/m^2^ (beginning of intervention)

### Exclusion criteria

- older than 70 / younger than 18 years
- meta-analysis
- intervention objective: reduction of electronic digital media consumption
- no direct intervention but through associated persons (social surroundings)

Control groups were accepted if they got the same intervention without support of digital media or a slimmed-down version, got the same intervention after the intervention group was finished (waiting list) or got no support at all. In case of a waiting list control group, cut-off criteria for inclusion was a maximum waiting time of 1 year.

Outcome variable was physical activity. Measured parameters of physical activity behavior include duration (MVPA min/day), intensity (e.g. METs/week or kcal/day), frequency (number of training units per day or week) and volume (e.g. steps/day or week or total PA) [29].

Search terms were “obesity”, “therapy”, “aftercare”, “intervention”, “digital media” and “physical activity” which were differentiated, specified and composed using the Boolsche operators. Therefore, 22,567 studies were identified in the first search. In a second, extended search, exclusion criteria were incorporated in the search strategy. Therefore 311 studies from PubMed, 418 studies from Web of Science and 467 studies from Cochrane Library were identified. Following the identification process, title and abstract of these studies were analyzed and duplicates removed. As a result, the total amount of studies was reduced to 109. In a last step, the remaining studies were analyzed in full-text, taking into account all inclusion and exclusion criteria. The identification process is shown in Fig. 1.

Approval by an ethic committee was not necessary because only published data were used.

For assessing risk of bias of the studies, the guidelines from Cochrane Germany [30] were used.

## Results

In total, 14 randomized controlled trials (evidence level 1a) could be included in this analysis (Table 1).

**Table 1:** Characteristics of 14 RCT’s included in the current systematic review

Study	Participants years/sex	Duration/FU (total)	Sample	Content		Control group
				Therapy	Aftercare	
Cussler et al. (2008) *USA* [21]	40–55 m/f	12 mo/− (12 mo)	*n* = 135 IG =66 CG = 69	–	(following a 4 mo weight loss program) support through internet via email, platform, chatrooms, website	no support
Donnelly et al. (2013) *USA* [26]	18–65 m/f	6 mo/ 12 mo (18 mo)	*n* = 295 IG = 201 CG = 194	Weekly meetings (approx. 60 min) via conference call	–	same meetings face-to-face (clinic)
Huber et al. (2015) *USA* [24]	18–55 m/f	3 mo/ 3 mo (6 mo)	*n* = 90 IG = 45 CG = 45	„portion control plate “+ telecoaching every second we, approx. 20 min (= 7 phone calls in total)	–	print materials
Hunter et al. (2008) *USA* [25]	Mean value 34 m/f	6 mo/− (6 mo)	*n* = 451 IG = 227 CG = 224	standard measure + computerized program (5x/wo online diary), weekly personalized feedback (online) + weekly topics on a website + 2 telephone conversations	–	only standard measure - > gym (minimum 3x/we), courses & preventive medical health check-up (face-to-face)
Kim et al. (2015) *Korea* [31]	20–60 m	6 mo/− (6 mo)	*n* = 205 IG = 104 CG = 101	4 meetings face-to-face (consultation) + daily personalized text messages	–	4 meetings face-to-face (consultation)
Markham Risica et al. (2013) *USA* [22]	18–70 f	7 mo/ 5 mo (12 mo)	*n* = 363 IG = 286 CG = 82	„SisterTalk “- > weekly interactive TV show + print materials	After Therapy ➔ 4 mo support via phone	waiting list print materials
McConnon et al. (2007) *UK* [32]	18–65 m/f	12 mo/− (12 mo)	*n* = 221 IG = 111 CG = 110	website, log in 1x/we (hints, tools & reminder via email, if no log-in was made)	–	print materials
Morgan et al. (2013) *Australia* [33]	Mean value 47.5 (SD 11.0) m	3 mo/ 3 mo (6 mo)	*n* = 159 IG1 = 54 IG2 = 53 CG = 52	„SHED-IT“ IG1 = DVD, print materials, support book IG2 = + website, online diary, 7 individualized feedback emails	–	waiting list
Pellegrini et al. (2011) *USA* [34]	21–55 m/f	6 mo/ - (6 mo)	*n* = 51 IG1 = 17 IG2 = 17 CG = 17	IG1 = Weekly group meetings. (3x) + individual consultation (1x/mo) (face-to-face), diary + BodyMedia Fit System (digital bracelet + display - > data upload website) + weekly written feedback IG2 = (only TECH): no meetings, same materials via email (1x/we) + BodyMedia Fit System +1x/mo Phone consultations	–	Weekly group meetings. (3x) + individual consultation (1x/mo) (face-to-face), diary
Rogers et al. (2016) *USA* [35]	21–55 m/f	6 mo/ - (6 mo)	*n* = 39 IG1 = 12 IG2 = 13 CG = 14	IG1 = no meetings, materials via email, BodyMedia FIT System (digital bracelet + display - > data upload website) + support via phone (1x/mo approx. 10 min) IG2 = + LINK activity Monitor (direct feedback via smartphone app)	–	Weekly meetings (30–45 min), print materials, diary
Routsalainen et al. (2015) *Finland* [36]	18–64 m/f	3 mo/ - (3 mo)	*n* = 46 IG1 = 15 IG2 = 16 CG = 15	IG1 = Facebook group (materials + consultation) + Polar Active physical activity (monitoring) IG2 = only Facebook group	–	Opportunity for face-to-face feedback after post measurement
Ströbl et al. (2013) *Germany* [37]	18–65 m/f	6 mo/ 6 mo (12 mo)	*n* = 467 IG = 228 CG = 239	–	following a 3 we obesity treatment (clinic) ➔ personal consultation (face-to-face) + 6 phone consultations (5–10 min)	Only 3 we obesity therapy no aftercare
Van Wier et al. (2009) *Netherlands* [38]	Mean value 43 (SD 8.6) m/f	6 mo/ - (6 mo)	*n* = 1386 IG1 = 462 IG2 = 464 CG = 460	IG1 = topics (phone consulting + phone consultation (every second week) IG2 = topics via interactive website + direct feedback via email (reminder via email or SMS, if no log-in was made)	–	phone consulting
Winett et al. (2007) *USA* [39]	Mean Value 53 (SD 13.9) m/f	3 mo/ 3 mo (6 mo)	*n* = 1071 IG1 = 322 IG2 = 330 CG = 298	IG1 = internet platform with 12 topics IG2 = + support via church	–	waiting list

- = not available, *m* male, *f* female, *mo* months, *we* week, *min* minutes, *n* sample, *IG* Intervention group, *CG* Control group, *approx..* approximately

Most studies were conducted in the United States of America (8). The remaining studies were from Australia (1), the Netherlands (1), Korea (1), Germany (1), Finland (1) and the United Kingdom (1). Most studies included both sexes (11), only two studies included just male participants and one exclusively included black women. The study periods varied from three to maximum 18 months. Most studies covered a time period of 6 months (7), followed by studies with a time period of 3 months (4). Follow-up data were collected in almost half of the studies (6). Sample size ranged from 39 to 1386 participants, with most studies in a three-digit range (10) and two studies with more than a thousand participants. Altogether, data from 4979 participants were analyzed for this review, 3043 of whom received an intervention assisted by digital media. More than half of the studies (8) randomized participants classically bilaterally into one intervention group and one control group. Six studies examined three groups with two intervention groups and one control group (see Table 1).

The digital media used for therapy in the trials werecategorized by author into four groups: cell phone/smartphone (calls, text messages and apps), computer (websites, platforms, emails and social networks), digital bracelet/pedometer and TV (programs and DVDs). Half of the studies (7) used only one digital device for intervention. Four trials used a combination of two different digital devices. The remaining three trials used more than two digital devices and were categorized as “multimedia” (Table 2).

**Table 2:** Digital devices used for obesity treatment in the 14 RCT’s included in the current systematic review

Digital devices	Amount
Phone/ smartphone	4
Computer	3
Phone/ smartphone & computer	1
Computer & pedometer/ digital bracelet	1
Computer & TV	1
Phone/ smartphone & TV	1
Multimedia	3
∑=	14

Most studies provided therapy, two of them only provided aftercare [21, 37] and only one study provided both [22] (see Table 1). Therapies were usually constructed modularly and addressed topics like dietary behavior, physical activity, behavior change strategies and overcoming barriers. Therefore, most online interventions created websites with personalized access for study participants, which allowed them to work independently on specific topics and to participate in discussion forums. In most cases expert advisors gave patients individual feedback by email or supported them by telephone regarding topics they worked on, answered questions, gave additional advice and provided information about the topics if needed or requested. Furthermore, coaching sessions on the phone focused on behavior changes, discussions on barriers, successes and failures and advice about patients’ action plans for the upcoming period [24]. Emails and text messages were used as automated reminders to “chase” participants who did not log in or upload their data within the agreed timeframe [32, 38]. Moreover, few studies used emails, text messages and phone calls to motivate participants (see Table 1).

One trial allowed participants to attend regular therapy sessions via conference call [26].

In the trials which used digital bracelets in therapy, participants received direct feedback about their activity from the display. Also after upload of the data by participants, expert advisors assessed the data with special software and gave participants individual feedback and additional guidance based on the analysis of their personal data [34–36].

One study used a public social network (Facebook) as communication platform [36]. Two studies integrated TV in their therapy to deliver content, one used an interactive TV show [22] and another one used a DVD to support their therapy [33].

In addition, two studies performed only media-assisted aftercare. One provided online support via email, platform, chatroom and a website [21] and the other provided face-to-face consultations via video calls [37].

The control groups mostly received the same material and information about diet, physical activity and positive behavioral change like the intervention group, but only in printed form and/or got face-to-face meetings or personal conversation with the therapist. Three trials applied the so-called “waiting list” method to their control group and two trials did not support their control group at all (see Table 1).

The analyzed studies used different kinds of instruments for measuring the physical activity outcome. Four studies used accelerometer-based measuring tools (BodyMedia FIT System (Jawbone); Polar Electro Kempele Oy; ActiGraph GT1X; ActiGraph GT3X; Actigraph LLC) and pedometer-based measuring tools (Yamax SW-200; WA101, Accusplit AE120). The majority of studies (10) used self-reporting measuring methods. Three of them used the International Physical Activity Questionnaire (IPAQ) [40], one of them the short form (IPAQ-SF) [41]. The remaining trials used the Seven-Day Physical Activity Recall Interview [42], the Paffenbarger Physical Activity Questionnaire [43], the Self-Reported Physical Activity and Screen Time Questionnaire [44], the Freiburg Questionnaire for Physical Activity [45], the Short Questionnaire to Asses Health-Enhancing Physical Activity (SQUASH) [46], the Beacke Physical Activity Questionnaire [47], and the Godin Leisure-Time Exercise Questionnaire [48]. One study did not specify the therein used self-reporting instruments [26]. Two studies used a combination of accelerometer based and self-reporting instruments [26, 36] (Table 3).

**Table 3:** Characterization of measuring instruments, parameters and outcomes in the 14 included RCT’s

Study	Measuring instrument(s)	Parameter	time-effect (PA)			time*group-effect (PA)
			IG 1	IG 2	CG	
Cussler et al. (2008) [21]	Seven-Day Physical Activity Recall Interview	kcal/day	↓	n.e.	↑	no
Donnelly et al. (2013) [26]	Self-report	PA min./week Steps/week	↑ ↓	n.e. n.e.	↑ ↑	no no
	Accelerometer	Counts/day	↓	n.e.	↓	no
Huber et al. (2015) [24]	IPAQ	total METs/week	↑	n.e.	↑	no
Hunter et al. (2008) [25]	IPAQ	total METs/week	–	n.e.	↑	no
Kim et al. (2015) [31]	IPAQ-SF	total METs/week	↑*	n.e.	↑	no
Markham Risica et al. (2013) [22]	Godin Leisure-Time Exercise questionnaire	Total Leisure Activity Score	↑	n.e.	↓	no
McConnon et al. (2007) [32]	Beacke physical activity Questionnaire	Score	n.d.	n.e.	n.d.	no
Morgan et al. (2013) [33]	Pedometer	steps/day	↑*	↑*	↑	yes*/yes*
Pellegrini et al. (2011) [34]	Paffenbarger Physical Activity Questionnaire,	kcal/week	↑*	↑*	↑*	no/no
Rogers et al. (2016) [35]	Paffenbarger Physical Activity Questionnair	kcal/week	↑	↑	↑	no/no
Routsalainen et al. (2015) [36]	Physical Activity Questionnaire (WHO)	days out of past 7 at least 60 min. of MVPA	↑	↑	↑	no/no
	Accelerometer	MVPA min/day	↑	–	↑	no/no
Ströbl et al. (2013) [37]	Freiburg Questionnaire for Physical Activity	h/week kcal/week	↑* ↑*	n.e. n.e.	↑* ↑	yes yes
Van Wier et al. (2009) [38]	SQUASH	Total PA (IQR) METmin./week	↑	↑	↓	yes/no
Winett et al. (2007) [39]	Pedometer	steps/day	↑	↑	↓	no/yes

*IG* Intervention Group, *CG* Control Group, *PA* Physical Activity, ↑ improvement, ↓ deterioration, − no change, * significant, *n.e.* not existing, *n.d.* no details

Studies also differed in terms of parameters that were used for objectifying physical activity (Table 3). Eleven trials found an increase in physical activity during the intervention period from baseline to the most recent measurement (time effect), four of them were significant. Furthermore, two studies found no differences during time and two studies reported a decrease in physical activity from baseline to final measurement. Control groups revealed similar results. Ten out of fourteen studies show an increase in physical activity between baseline and final measurement, three of them were significant. Four studies reported a decrease in physical activity in their control groups and one study did not find any time effect, neither for the intervention group nor for the control group. Reported results regarding the time*group effect are relatively homogeneous. Four out of fourteen studies were able to prove a positive effect, two of them only partial. Only one study showed a significant time*group effect in increasing physical activity [33]. The twelve remaining studies report no significant time*group effect when comparing the intervention and the control group during the intervention period (Table 3). Only two studies recorded their effect size. Morgan et al. [33] report a moderate effect (Cohen’s *d*) and Ströbl et al. [37] report a small effect (*ƞ*^*2*^) [49].

In addition, most of the studies were also collecting and analyzing anthropometric data to calculate the Body Mass Index (BMI) as well as lifestyle-relevant behaviors like dietary behavior.

### Limitations

In the course of the implemented risk of bias assessment in this systematic review, none of the identified studies need to be excluded due to excessive risk of bias.

Regarding the results of this systematic review, the authorship would like to point out a few limitations. Some studies do not report information about significances of the results regarding the time effects within their study groups. Time*group effects were reported in all studies, mostly stating *p*-values. Also some studies deemed their results to be significant, however did not provide any numerical values to support their claim. Further limits are missing data about effect size, since just two trials indicate their effect size at all. This strongly limits the interpretation of practical relevance.

In this presentation of results (Table 3) the last measuring points (follow-up data, if available) were continuously used for analysis. This leads to an extension of the study period by three to 18 months after baseline, depending on the study, and limits comparability. Just a few studies report significant time effects and time*group effect directly after intervention. Moreover, these few significances mostly disappear after follow-up. One explanation is the dwindling effect of the intervention.

Another limitation concerns validity as different measuring instruments were used to measure the physical activity level, e.g. different types and generations of accelerometer- or pedometer-based instruments by different providers. Also the self-reporting measuring instruments differ in content, quality and context (e.g. profession, leisure time, sports club etc.).

The clinical recommendations given in the following discussion are also limited due to lack of information about the therapy content. Nearly no information was provided or at least very short and limited explanations were given making it difficult to compare therapeutic approaches and, thus, limit the interpretation.

## Discussion

Overall, this systematic literature shows a positive effect of media usage on physical activity levels in obesity treatment, taking into account the above-mentioned limitations. Physical activity in the intervention groups, measured by either duration, intensity, frequency or volume, increased in eleven trials and decreased in the other three trails. Comparable results can be found for the control groups (see Table 3). Accordingly, therapeutic approaches using digital media in obesity treatment did not show superior benefit over traditional therapeutic methods like face-to-face meetings of patient and therapist or the provision of printed (information) material.

The present analysis identified different study concepts. In most studies (8), the control groups received the same information as the intervention group only in written (print material) or verbal (face-to-face) form. Six studies did not support their control group at all during the intervention period. In three of those six studies the control group had the opportunity to participate in the full intervention after the end of the study (waiting list). One of those six studies applied a mixed design [22], i.e. the control group received little support during the intervention period and was added to the waiting list after the study. Interestingly, the latter study [22] shows a decrease in physical activity in the control group and an increase in physical activity in the intervention group. This study is also the only one which used various digital media in both, treatment and aftercare. There was no time*group effect. The increasing physical activity in the intervention group in contrast to the decreasing physical activity in the control group indicates a working concept for obesity treatment. In the other two studies [38, 39] with the same results, the time*group effect is only partially present and not significant. In total, four studies were found in which physical activity level increased in the intervention group compared to the control group. Only one study [33] shows a significant time*group effect.

All studies reporting the desired effect of increased physical activity have one thing in common: All use a combination of digital media support and face-to-face consultations. This fact indicates that this mixed design is advisable in obesity treatment.

Within its limitations, practical recommendations for obesity treatment can be derived from the results of this systematic review. First, it is advisable to continue to integrate therapeutic personnel in obesity treatment and not to exclusively use digital media.

For example, in outpatient settings that commonly include less time for personal therapeutic support, the media-assisted approach is useful to increase physical activity in obesity patients. Here a simultaneous use of various digital media seems to be effective to address important aspects of behavior, e.g. nutrition and physical activity. However, it is still unclear if the used media need to be consumed on specific devices or rather in a certain combination of devices. For this purpose, more research is required [50]. As an example for a successful combination of digital media serves the study of Morgen et al. [33] – the only one with a significant time*group effect. It employed a combination of various online services with information about dietary and physical activity behavior, a DVD with practical exercises and a personal online diary that was used to provide personalized feedback by email [33].

In rural areas access to and accessibility of adequate therapy centers is mostly challenging and partly difficult to implement. In this case, media-assisted treatments and – particularly – aftercare has the potential to increase physical activity levels and, thus, contribute to the success of obesity therapy [26].

Furthermore, digital intervention concepts were well received by patients and seen as very helpful and supportive (see also [31, 37, 51]). This could be attributed, among other reasons, to the time saving aspect because of reduced travelling distances. In addition, digital therapy concepts can help to reduce barriers to allow and increase therapy participation, especially in rural areas [26].

Another important aspect of weight loss is self-monitoring and this, in turn, can be supported simply by using digital devices for motivation [32]. However, personal care in obesity treatment is essential to achieve the intended effect.

From an economic perspective, digital media could offer a low-cost alternative in the long-term. Even if overall costs briefly increase due to development of adequate software and procurement of digital devices, they will decrease in time for the healthcare systems and the patiens as costs for human resources and travel expenses decline [26, 35, 52].

## Conclusion

In view of the increasing digitalization of our lifes, it seems only natural that media-assisted obesity treatment will increasingly become an integral part of obesity research and therapy [34]. The evidence in this issue is still unclear. This review of 14 randomized controlled trials (RCT) showed the greatest potential for a therapeutical approach using digital media for supporting obesity treatment in combination with a traditional face-to-face treatment. This result confirms already existing research in this field [53].

Nevertheless, major randomized controlled trials are necessary to identify effective methods for helping obese patients in the long term [36]. Existing studies provide initial indications on how to achieve intervention objectives and increase physical activity by using digital media. Through this, it is possible to implement efficient and resource-conserving concepts of intervention for both, the healthcare system and patients in the future. In order to build on existing success, adequate evaluation and further development of media-assisted obesity treatment and aftercare is required.

## Data Availability

Data will be available upon request of the corresponding author.

## Abbreviations

BMI: Body Mass Index
MET: Metabolic Equivalent of Task
MVPA: Moderate to vigorous physical activity
PA: Physical Activity
PICO: Population, Intervention, Comparison, Outcome
PRISMA: Preferred Reporting Items of Systematic Reviews and Meta-Analysis
RCT: Randomized Controlled Trials
